# Ambiguous loss in family caregivers of patients in a permanent vegetative state: A reflexive thematic analysis

**DOI:** 10.1017/S147895152610282X

**Published:** 2026-06-15

**Authors:** Ines Testoni, Matteo Rigo, Laura Pizzolato, Adriana Frenda, Ciro De Vincenzo

**Affiliations:** 1 Department of Philosophy, Sociology, Pedagogy and Applied Psychology (FISPPA), University of Padova, Padova, PD, Italy; 2 Department of Political Science, Law and International Studies, University of Padova, Padova, Italy; 3 Permanent Vegetative State Unit, Institute of Care Services “Cima Colbacchini” (ISACC), Bassano del Grappa, Italy

**Keywords:** Ambiguous loss, permanent vegetative state, family caregiving, reflexive thematic analysis, identity transformation

## Abstract

**Objectives:**

Family caregiving for individuals in a permanent vegetative state (PVS) represents an extreme form of ambiguous loss characterized by prolonged emotional strain, suspended grief, and profound identity disruption. Despite its complexity, few qualitative studies have examined how families navigate this uniquely challenging condition and construct meaning around it.

**Methods:**

Adopting a phenomenologically informed, interpretative approach, we conducted semistructured interviews with 13 family caregivers (in northeastern Italy). Data were analyzed via reflexive thematic analysis, following Consolidated Criteria for Reporting Qualitative Research (COREQ) guidelines.

**Results:**

Four interconnected themes emerged: (1) “the paradoxical coexistence of presence and absence”; (2) “identity disruption and emergent growth”; (3) “bodily relational practices that sustain emotional bonds and resist biomedical finality”; and (4) “the ‘double face’ of waiting and farewell, spanning ontological and biological loss.” Caregivers described symbolic acts that maintain relational continuity and challenge dominant medical framings of the patient’s body.

**Significance of results:**

Ambiguous loss in PVS should be understood as a dynamic relational and symbolic process rather than a static burden. Effective support must honor families as active meaning-makers. Clinical practice and policy should integrate narrative and relational ethics to mitigate prolonged grief and enhance caregiver resilience.

## Background

The prolonged illness of a loved one exerts profound emotional, relational, and psychological effects on family caregivers (Lim and Zebrack 2004; Schulz and Sherwood 2008). Caregiving for relatives in a permanent vegetative state (PVS) or minimally conscious state is particularly burdensome (Cipolletta et al. 2016; Bulauan et al. 2023). A central framework for understanding this complexity is ambiguous loss (Boss 1999, 2004), describing a state where a loved one is physically present but psychologically absent. This unresolved grief can evolve into prolonged grief disorders, especially when social recognition and psychological support are lacking (Giovannetti et al. 2013).

Beyond emotional distress, caregivers face financial strain and disrupted family dynamics. Many undergo a significant identity transformation – shifting from original family roles to full-time caregivers – often resulting in “role engulfment” and social isolation (Montgomery and Kosloski 2009). The absence of advanced directives further complicates ethical decisions regarding life-sustaining treatments (Kitzinger and Kitzinger 2015). Despite poor clinical prognoses, caregivers often interpret minimal reflexive gestures as manifestations of presence – a process of reflective functioning where intentionality is attributed to the patient’s body to preserve relational agency (Fonagy and Target 2002; Zulato et al. 2023).

Gender and spousal status influence these dynamics; male caregivers appear more vulnerable to anxiety and depression (Chiambretto et al. 2001), while female spouses often experience a “liminal” state of simultaneous love and despair (Hamama-Raz et al. 2013). While research predominantly focuses on burden, growing attention is turning to posttraumatic growth (PTG), the positive psychological change emerging from traumatic events (Tedeschi and Calhoun 2004). However, PTG depends on narrative meaning-making and reflective processing. This study addresses the limited qualitative research on how PVS caregivers negotiate these evolving identities and develop strategies for emotional resilience.

## Methods

### The present study

This qualitative study uses a phenomenologically informed, interpretative approach to explore how family caregivers of individuals in a PVS emotionally adapt to long-term care. Grounded in ecological-hermeneutic and psychosocial paradigms (Geertz 1973; Frosh 2003), the research addresses the paucity of data in the Italian context. It focuses on the relational and symbolic dimensions of nonreciprocal care, contributing to broader discussions on ambiguous loss and identity transformation.

### Methodology and research design

Following standards (Tong et al. 2007), the study employs reflexive thematic analysis (RTA) (Braun and Clarke 2006, 2019). This epistemological orientation conceptualizes participants as active, interpretive agents (see Table 1).

**Table 1.** Overview of study design and procedures Table 1 long description.

Phase	Description
Study aims	To investigate the psychological and relational impact of caring for a family member in a permanent vegetative state (PVS).
Inclusion criteria	Participants aged ≥18 years; primary caregivers (past or present) with a minimum of 6 months’ caregiving experience; provided informed consent.
Participant recruitment	Final sample: *N* = *13* (*7* females, *6* males); purposive and snowball sampling through caregiver associations and community networks.
Pilot testing	Three pilot interviews conducted to refine the semistructured interview guide; pilot data excluded from the final dataset.
Semistructured interviews	Conducted via Zoom, audio-recorded, and transcribed verbatim; original interviews in Italian, translated into English.
Verbatim transcription and translation	Transcripts checked for accuracy and consistency; translation verified by bilingual researchers for semantic fidelity.
Reflexive thematic analysis	Primary researcher: initial coding and analytical memos; secondary researcher: cross-checking and peer debriefing; external reviewer: feedback on theme development.
Trustworthiness procedures	Themes reviewed and validated by 2 independent judges to ensure credibility, confirmability, and analytical rigor.
Results report	Final thematic structure prepared, integrating illustrative participant quotes and narrative extracts to enhance transparency and coherence.

RTA emphasizes the researcher’s reflexive engagement, utilizing subjectivity as an analytic resource to co-construct meaning from the complex lived experiences inherent in disorders of consciousness.

### Data collection methods

Semistructured interviews served as the primary data collection method, balancing a framework based on prior literature with openness to emergent content. The interview guide was refined through 3 pilot interviews with field professionals to ensure thematic coherence and emotional accessibility. Data collection occurred between June and September 2024 via Zoom. Interviews lasted 60–90 minutes, were audio-recorded, and transcribed verbatim. Transcripts were translated from Italian to English with native-speaker assistance to ensure semantic fidelity.

Ethical approval was granted by the Research Ethics Committee of the University of Padua (protocol no. 225-d). The study was conducted in accordance with the Declaration of Helsinki and APA ethical standards. Participants provided electronic informed consent, and data were managed per the European General Data Protection Regulation (GDPR) (Regulation EU 2016/679). The team remained prepared to provide psychological support; however, no adverse reactions were reported.

### Participants

Using purposive and snowball sampling (Etikan et al. 2016), the study recruited family caregivers with a minimum caregiving duration of 6 months. The final sample consisted of *N* = 13 participants (7 women, 6 men), including spouses, siblings, and adult children (mean age: 57.7; see Table 2).

**Table 2.** Demographic characteristics of family caregivers participating in the study (*N* = 13) Table 2 long description.

Fictitious name ^a^	Gender	Age	Degree	Employment	Relation to patient	PVS duration	Status
A.F.	Male	59	No formal education	Retired	Wife	32 years	Deceased
A.G.	Male	32	PhD candidate	Professional geologist	Father	2.5 years	Deceased
B.M.C.	Female	57	High school diploma	Sales clerk	Husband	5.5 years	Deceased
C.G.	Male	43	High school diploma	Sales assistant	Mother	1 year	Deceased
E.F.	Female	50	High school diploma	IT technician	Mother	2.5 years	Deceased
G.B.	Male	42	High school diploma	Insurance employee	Sister	23 years	Deceased
M.B.	Female	53	High school diploma	Office worker	Father	14 years	Deceased
R.R.	Male	74	No formal education	Retired	Wife	19 years	Currently in PVS
R.S.	Female	65	High school diploma	Administrative assistant	Husband	3.5 years	Deceased
R.B.	Female	73	No formal education	Retired	Daughter	23 years	Deceased
S.B.	Male	75	No formal education	Retired	Daughter	23 years	Deceased
V.L.	Female	72	Graduate	Retired	Husband	3 years	Deceased
S.R.	Female	55	High school diploma	Administrative assistant	Husband	1 year	Deceased

*Note*: Pseudonyms are used to protect participants’ anonymity.

To protect participant confidentiality, each participant was assigned a pseudonym, which was used throughout the transcripts and the final reporting. Sample adequacy was evaluated through the principles of information power and saturation. A saturation threshold emerged after the tenth interview and was confirmed by the thirteenth, focusing on the depth and diversity of lived experiences rather than statistical generalizability.

### Data analysis

Following the recursive 6-phase framework of RTA (Braun and Clarke 2006, 2022), the analysis involved: familiarization, code generation, theme development, review, naming, and reporting. A hybrid inductive–deductive strategy was employed using ATLAS.ti (version 25), allowing for both theoretical sensitivity and empirical openness. The first author led the initial coding, maintaining analytic memos to document evolving interpretations.

The analysis was an iterative process; regular discussions with the second author provided an external perspective, enhancing critical reflection (Braun et al. 2019). Relevant text segments were labeled, reviewed for patterns, and grouped into “families” of codes. These categories supported the development of higher-order themes reflecting participants’ lived experiences within broader relational contexts. Throughout, RTA was treated as a creative practice, acknowledging that qualitative data are simultaneously subjective and intersubjective.

#### Reflexivity and positionality

A reflexive stance was maintained to account for how the research team’s backgrounds shaped data interpretation (Finlay 2002). The first author utilized an empathetic, nonjudgmental approach during fieldwork, while the second author provided a distanced analytical perspective to surface less explicit meanings. Participants were viewed as co-constructors of knowledge, and their narratives as epistemic acts that challenge dominant representations of agency and presence in medicalized settings. This design reflects a commitment to a transdisciplinary psychology attentive to the symbolic and ethical dimensions of situated knowledge.

## Results

From the analysis of the narratives, 4 main thematic areas emerged: “Presence in absence and absence in presence,” “Psychological readjustment: being thrown into the world,” “Bodily relational lived experiences: confronting the body,” and finally, “The next: the ‘double face’ between waiting and departure” (see Table 3).

**Table 3.** A summary of the themes, definition, and example of quotes Table 3 long description.

Themes	Definition	Examples of quotes
Presence in absence and absence in presence	Describes the **paradoxical coexistence** of physical presence and psychological/emotional absence, fragmenting identity recognition and creating relational liminality. Highlights how minimal bodily responses are resignified to sustain symbolic continuity.	“My sister was no longer my sister … it was like talking to a plant.” “Yet sometimes when I held his hand, the way he squeezed it was different … for me he recognized me.”
Psychological readjustment: being thrown into the world	Captures the sudden **existential and practical reconfiguration** caregivers undergo, echoing Heidegger’s concept of *Geworfenheit* (*thrownness*). Includes both the **material burden** (new responsibilities) and the **identity transformation** that emerges.	“It’s a piece of you that has gone away. Your life is never the same as before.” “I had to learn to do everything he used to handle; it was like becoming another person.”
Bodily-relational lived experiences: confronting the body	Explores the **ambivalence** between cognitive awareness of irreversible loss and the affective impulse to maintain connection through **bodily care rituals** (touch, grooming). The patient’s body becomes a **site for sustaining hope and symbolic presence**, challenging biomedical finality.	“I dressed him, I combed his hair, I changed him. I still believed he could hear me.” “A tear would fall … for us that was a sign he was still there.”
The next: the “double face” between waiting and departure	Describes the **suspended temporal dimension** in which caregivers negotiate the coexistence of *ontological death* (loss of relational reciprocity) and *biological death*. Shows how grief becomes a **fragmented, nonlinear process**, balancing waiting, ambiguous hope, and eventual release.	“When he died, I felt that a part of me died with him. But at the same time, the suffering finally had an end.” “I could not say goodbye completely, even when it was clear he would never come back.”

## Presence in absence and absence in presence

A central theme is the complex dialectic between “presence in absence” and “absence in presence.” In PVS, identity recognition disintegrates: the person is physically present, but the traits defining individuality – voice, conscious gaze, and agency – seem to vanish. These physical changes lead family members to perceive a profound absence, where the altered body appears distant from the person they knew. V.L. shares this regarding her husband:

He became another person, a disfigured person, not in that sense, but anyway. It was not him. There was nothing left, nothing more; there was this vegetable, who breathed occasionally. However, no, I never had the feeling he could improve; he was, I repeat, a vegetable. With these eyes that no longer saw, the mouth that occasionally made involuntary movements.

This fracture forces caregivers to redefine both the patient’s identity and the meaning of the bond. G.B. recounts the unique pain of absence perceived within a physical body, leading to a breakdown of relational will:

My sister was no longer my sister, for me she was there, after a while you do not even have that will to go there and talk to her because you realize afterwards, that there was no relationship […] I did not interact with her anymore because for me she was not there, it was like talking to a plant.

The persistent condition weakens familiarity, with suffering stemming from the loss of a recognizable gaze and reciprocity. M.B. emphasized the struggle of the later years when the patient appeared increasingly catatonic:

The last two years we were also truly not very present; at a certain point these states stabilize in their state of being and therefore become increasingly catatonic. However, even from dad there was no response and therefore the last three years I actually struggled even to see him.

Conversely, many participants reported perceiving presence through bodily movements. R.S. insists that facial expressiveness remains a vital link:

My husband had preserved expressiveness in his face. In addition, even there everyone says no, but they are grimaces because they are in a vegetative state like that. However, I assure you that it’s not true, because when I stayed there … I spent those three quarters of an hour giving him foot massages, trying to move those rigid arms, putting creams on his skin and he made it understood that he didn’t like it.

In this modality, closeness becomes a silent dialogue of care and minimal signals. C.B. shared how these physical responses allowed her to maintain recognition:

I always tried to tell him beautiful things […] I felt the touch of his hand, for me it changed. They said it was an instinct that when you put your hand there, they squeeze it, but the way he squeezed it was different sometimes, when I held his hand and told him certain things […] And afterwards, in my opinion, he understood that it was us. He recognized me, yes.

Finally, some found continuity through moments of perceived lucidity or within the spiritual realm. A.F. noted:

she had moments of lucidity […] she listened to you […] there were certainly reactions to our presence,

while S.R. felt closeness through dreams. These experiences show how “loss” and “presence” alternate, forcing a precarious balance between maintaining the bond and accepting its transformation.

## Psychological readjustment: Being thrown into the world

Participants’ narratives reflect the Heideggerian concept of “being thrown into the world” – suddenly finding oneself delivered to undetermined conditions. The PVS event was an irruption of contingency that delivered families to an unexpected reality. Despite its destructive nature, this “thrownness” possessed generative power. E.F., who became his mother’s legal guardian, recounts mastering this role to eventually support others:

Let’s say it’s a truly comprehensive experience from the medical to the bureaucratic point of view, because even the matter of legal guardianship, of the court, is not an easy thing, I did it completely independently. Now, more than one person has asked me for help, and I have managed, owing to the experience, to give a great hand to others who needed it. This is already a great satisfaction.

For many, pain and loss demolished identity balances, forcing a redefinition of the self. R.B. recounts how caring for her daughter restricted her freedom and shifted her life’s priorities:

Well, naturally many things changed, because for the first 10 years I was always at home. I never detached myself; I only went out on Saturday mornings to do the shopping, and my husband stayed with her […] It’s something that, even though many years have passed, is a piece of you that has gone away. Your life is never the same as before.

Beyond material changes, caregivers experience a profound reassembly of identity. S.R. had to assume complex responsibilities previously managed by her spouse:

And so the difficulty of moving forward has been there and still is. However, I had to learn to do all those things that he did and that I didn’t do […] I took care of children, family […] he had the bureaucratic part, the financial part of the family. Therefore, I took all those things for granted; he did them. I was never interested. In addition, I found myself having to learn to do everything.

However, this transformation can instill a profound sense of unease. G.B. describes how trauma generated a constant state of tension regarding his daughters:

I’m not living fatherhood in the best way, because I always have a bit of this anxiety towards my daughters; so, sometimes maybe I see that she looks out the window, I make up the story that she falls from the window and dies or remains in the same condition as my sister. So I often make up all these stories […] you always have this state of anxiety a little bit towards their health and their well-being.

C.B. similarly recounts how the experience stripped away her ability to plan, leaving her in a state of reactive nervousness:

I’ve become a bit colder, I can’t make plans anymore … I cannot say well, in six months I’ll go here, I’ll go there […] I can’t plan things long-term; I try to live my day in the best possible way […] I snap more easily, I often become maybe sadder. I’m a bit more nervous, I respond badly even at work or to my children.

Finally, in the void left by loss, R.R. approached sports as a lifeline, though his account reveals a reckless disinterest in danger:

Therefore, I threw myself into sports, and that’s what saved me […] you try to think as little as possible about all the problems […] I think I’ve risked my life 3, 4 times, but there’s nothing to laugh about […] I often don’t know, I run stop signs without thinking about it much, etc., what the fuck do I care, I live like this period and that’s it, if I have to go I go end.

Ultimately, being “thrown” into tragedy forces the individual to redefine the self and find a new way of existing.

## Bodily relational lived experiences: Confronting the body

Caregiving in PVS is marked by a contradiction between cognitive awareness and the affective sphere. Rationally, medical communication excludes recovery, yet this knowledge cannot eradicate the emotional bond. The body becomes a site of tension between accepting the end and refusing separation. A.F. reflects on how the emotional bond persists despite minimal clinical hope:

The doctors always gave me very little hope, but I became somewhat attached to faith. I was very Catholic; I believed in it a lot. Well, the emotions were always strong, also because you know, at the beginning the breathing, the tear, because this tear also fell. Sometimes you would tease him, and he would move. For us, these were very important signs.

Manifestations like breathing or tears are symbolized as signals of life, acting as a defensive mechanism that delays the acceptance of “affective death.” Continuous care becomes a psychological strategy to avoid awareness of loss. S.R. describes how hope intertwines with the need for care:

I dressed him, I combed his hair, I changed him. I wanted him to always be as beautiful as possible. Yes, I still believed in it. I believed he could hear me. I always had a bit of hope. I could not detach myself; I refused to think that it was not him anymore.

These acts are symbolic rituals conferring identity onto the body. Physical interaction becomes the last form of communication, fueled by hope. However, this conflict can trigger “affective isolation” as family members engage in differing acceptance processes. M.B reflects on this disparity:

For me, it was difficult to accept that he could no longer be there, but my sister, my mother, had already put themselves in the idea of no longer having him; it was just as if he were dead … I, however, could not do it.

Despite the absence of awareness signals, family members seek a recognizable presence. E.F. describes this internal hope:

I was convinced that he definitely perceived something. I had already put myself in the idea of the funeral and these processes, but inside me, I continued to hope for something different. I will remember some scenes charged with hope that are impossible to forget.

The body becomes a “borderland” where physical gestures attempt to evoke a reaction. C.G. recounts using the body as a medium for connection:

I caressed my mother, I tickled her […] and she had a reaction; she moved her foot.

Yet, this desire often meets the frustration of silence. R.R. emphasizes the difficulty of this one-sided communication:

When you see her like that, with her eyes closed, her mouth open […] it’s difficult. If you talk to her, you try to communicate, but in the end, nothing changes. There’s not much to do. Unfortunately, even if you try to tell her something, it is not what will solve the situation.

Ultimately, the PVS experience is an interweaving of rationality and affection. The body remains fertile ground for emotional resistance, where caregivers seek to maintain a connection that challenges definitive separation.

## Next: The “double face” between waiting and departure

The concept of “the next” represents a temporal dimension oscillating between ontological and biological death. These phases intertwine, creating a suspended space where waiting and departure coexist. Ontological death refers to the loss of relational essence, whereas biological death marks the physical loss, often resulting in definitive closure or relief.

### Ontological death: An interrupted monologue

Ontological death is a phase of “absence–presence” where communication becomes a unidirectional monologue. This generates a suspended pain, as seen in M.B.’s account:

I knew that my father wasn’t truly with us, but I could not resign myself to the fact that he would never come back, that I could never talk to him like before. It was as if his presence was still present, but it was intangible. We still needed to hope, to believe that a miracle could happen.

This grief involves a fracture between biological reality and emotional perception. V.L. reflects on the difficulty of letting go:

At the moment when he no longer reacted […] I could still not let go. I could not say goodbye completely … It was as if I had to get used to this separation without him truly allowing me to.

For R.R., the loss of reciprocity generates deep loneliness:

certain situations present themselves again, but now you find yourself alone, without her. You have to think […] just to remember, to see how you could solve certain situations.

Caregivers adapt through various mechanisms. A.G. took refuge in professional continuity:

when dad’s accident happened, I started doing his job. I carried on the company.

Conversely, S.B. experienced a crisis of faith:

it’s as if I’ve had entire months of intense disappointment, a certain anger, especially toward my religion and toward God.

## Biological death: The plurality of the “after”

Biological death represents the definitive caesura. The “after” is an interweaving of relief, acute pain, and late acceptance. For many, physical death marks a necessary liberation from years of uncertainty. R.S. recounts:

When he died, I felt that a part of me died with him. However, at the same time, his body was no longer there, and this made me feel that finally, the suffering that had lasted for years had ended. It was a relief but also an enormous emptiness.

This emptiness is often triggered by anniversaries or memories. Physical separation is tangible, yet emotional detachment remains elusive years later. A.F. declares:

Years later, I still have never accepted this thing.

S.R. describes the nonlinear nature of this process:

It’s not that I’ve gotten used to his absence, I’m learning to accept that he’s not there […] but there’s no day that I do not think about it.

C.B. highlights the ongoing need for support to find stability:

I’m still getting help because I had periods when I seemed to be doing well […] I decided it was better to let everything go and live my life. I started to appreciate my solitude.

Ultimately, physical death makes the separation final. M.B. concludes:

In the end, when it happened, I understood that it had to happen like this […] but I wasn’t prepared to not be able to see her anymore. Physical death made me understand that the separation was definitive. There was nothing more to expect, except pain.

“The next” is thus a fragmented journey between memory and the search for new meaning, a process that continues long after the physical departure.

## Discussion

This study examines family caregiving in PVS as an evolving process interweaving ambiguous loss, identity disruption, and embodied relationality. By adopting a psychosocial lens, we move beyond the “individual burden” perspective to highlight how symbolic and relational dimensions shape caregiver resilience and sense-making.

### Ambiguous loss as a relational and symbolic process

Findings indicate that caregivers experience ambiguous loss not as a static event but as an evolving process (Boss 2004; Sawicka 2017). PVS entails a paradoxical co-presence of physical continuity and psychological absence (Chiambretto et al. 2001; Pinel-Jacquemin et al. 2021; Testoni et al. 2023). Participants interpreted minimal reflexes as intentional acts, resignifying signs as evidence of volition to maintain emotional bonds (Neimeyer et al. 2006; Stroebe et al. 2010; Zulato et al. 2023). While ambiguous loss is a known predictor of prolonged grief and psychological distress (Boss 2010; Hollander et al. 2016; Küçükkaragöz and Meylani 2024), our study shows that meaning-making is also a creative act. Caregivers use it to sustain relational continuity, resist biomedical narratives of finality, and preserve the symbolic identity of the loved one (Valentine 2019).

### Identity disruption, role engulfment, and emergent growth

Consistent with Heidegger’s *Geworfenheit* (thrownness), caregivers were abruptly cast into unfamiliar existential terrain without institutional support (Heidegger 1962; Giovannetti et al. 2012; Huber et al. 2012). This existential thrust mirrors research on identity disruption in chronic contexts (Stawnychy et al. 2021; Choi et al. 2024). Preexisting family roles dissolved into an engulfing caregiving identity – a pattern documented in acquired brain injury and dementia (Etters et al. 2008; Cheng 2017). However, the data also underscore a potential for PTG (Tedeschi and Calhoun 2004); caregivers described developing bureaucratic, medical, and advocacy skills, finding a deepened purpose in supporting others. This duality highlights the need for interventions that recognize both resilience and enduring vulnerability to prevent isolation (Adelman et al. 2014; Lambert et al. 2016).

### The liminal body: Between objectification and relational agency

A distinctive contribution of this study is its focus on the body as a liminal space. Routine acts – massaging limbs, styling hair, or speaking to the patient – become symbolic rituals sustaining the affective bond (Butcher and Buckwalter 2002; McAndrew et al. 2023). These practices challenge biomedical framings of the body as “vegetal” (Kitzinger and Kitzinger 2018), revealing the body as a “site of memory and hope” (Havelka et al. 2009; Bolton and Gillett 2019). Similar to dementia care, where aesthetic practices maintain dignity (Giebel et al. 2021), PVS families act as co-constructors of embodied personhood, resisting the finality of medical prognoses.

### Temporal suspension and “double death”

Participants described how ontological death – the loss of relational agency – precedes biological death, creating a state of “living bereavement.” This aligns with Boss’s (2004) theory that absence is cyclical rather than linear. Without institutional recognition, this suspended mourning exacerbates anxiety and moral distress (Mendenhall and Boss 2022). These dynamics correspond with literature on post-intensive care syndrome – family (PICS-F) and ambiguous loss in intensive care unit (ICU) settings (Imanipour et al. 2019; Zante et al. 2020; Smith et al. 2025). Our findings suggest that PVS contexts challenge normative conceptions of grief, invoking the concept of continuing bonds as a transformed connection to the individual (Klass 2006; Testoni et al. 2022).

### Toward relational ethics and care practices

These findings necessitate expanding the focus from burden toward understanding caregivers as relational agents. Families negotiate ambiguity through symbolic practices that sustain dignity and agency. Clinical training and policy frameworks must move beyond instrumental support to integrate narrative and creative dimensions that honor the complexity of lived experience (McKenna et al. 2022; Nia et al. 2022; Reckrey et al. 2022). Recognizing families as epistemic agents is a moral imperative that challenges our understanding of connection and identity when life unfolds between presence and absence.

## Conclusion

This study illuminates the relational, existential, and symbolic dimensions of caregiving for individuals in a PVS. By foregrounding caregivers’ voices, it expands the discourse on ambiguous loss, identity transformation, and embodied relationality. It underscores the necessity of viewing caregiving not merely as an individual burden but as an intersubjective process situated within complex moral, cultural, and institutional contexts.

### Limitations and directions for future research

The research is limited by its geographic scope, involving 13 participants from northeastern Italy, which may not reflect the diversity of global socioeconomic or legal attitudes toward life-sustaining treatment. Furthermore, the cross-sectional design cannot capture longitudinal shifts in coping trajectories. Future research should compare PVS caregiving with other contexts, such as dementia or ICU survivorship, to identify divergent patterns of ambiguous loss. Additionally, quantitative or mixed-methods studies could map the prevalence of prolonged grief and resilience factors over time to complement these qualitative findings.

### Implications for practice and policy

Different practical and policy implications were pointed out by the different insights gained. First, there is a need for specific training for healthcare professionals in ambiguous loss and relational liminality. Also, psychosocial support should integrate narrative and creative methods to validate “continuing bonds.” Finally, institutional frameworks must incorporate relational ethics into clinical decision-making, while policies should establish formal counseling and peer networks to prevent caregiver isolation.

## Data Availability

The datasets generated and analyzed during the current study are not publicly available to protect participant confidentiality but are available from the corresponding author on reasonable request and with the participants’ consent.

## Table 1 Long description

The table outlines the phases and procedures of a qualitative study examining the psychological and relational impact of caring for a family member in a permanent vegetative state. Eligible participants were adults aged eighteen or older who were primary caregivers, past or present, with at least six months of caregiving experience and who provided informed consent. Thirteen caregivers participated, including seven women and six men, recruited through purposive and snowball sampling via caregiver associations and community networks. Three pilot interviews were used to refine the semi-structured interview guide, and the pilot data were not included in the final analysis. Interviews were conducted on Zoom, audio-recorded, transcribed word for word, and translated from Italian to English, with bilingual verification to preserve meaning. Analysis used reflexive thematic analysis with initial coding and memos by the primary researcher, cross-checking and peer debriefing by a secondary researcher, and feedback from an external reviewer on theme development. Trustworthiness was strengthened by having two independent judges review and validate themes for credibility and confirmability. The results were reported as a final thematic structure supported by participant quotes and narrative extracts, noting that findings reflect a small, purposively recruited sample.

Navigate back to Table 1.

## Table 2 Long description

The table lists 13 family caregivers by pseudonym, describing gender, age, education, employment, relationship to the patient, how long the patient remained in a persistent vegetative state, and the patient’s status. Ages range from early thirties to mid seventies, with many caregivers in their sixties and seventies. Education is most often a high school diploma, with several reporting no formal education and one listed as a PhD candidate. Employment is frequently retired, especially among the oldest caregivers, while others work in roles such as administrative assistant, office worker, IT technician, insurance employee, sales, and geology. Relationships to the patient include spouses, parents, children, and a sibling, with spouses and parents appearing most often. PVS duration varies widely from about one year to more than three decades, with multiple cases around two to three years and several very long durations around fourteen to thirty two years. Patient status is deceased for all but one case, where the patient is still in PVS.

Navigate back to Table 2.

## Table 3 Long description

The table organizes four qualitative themes about caregivers’ experiences, pairing each theme with a definition and sample participant quotes. Theme one describes a paradox where the person is physically present but emotionally absent, with small bodily responses reinterpreted as recognition. Theme two focuses on abrupt life disruption and identity change as caregivers take on new responsibilities and feel life is permanently altered. Theme three centers on intimate care of the body, where grooming, touch, and noticing tears are used to sustain connection and hope despite awareness of loss. Theme four highlights a suspended time of waiting and gradual departure, where grief is fragmented and mixes relief at the end of suffering with an inability to fully say goodbye. Quotes in each row serve as illustrative examples rather than counts or prevalence, so the table supports thematic understanding rather than statistical comparison.

Navigate back to Table 3.
